# OPETH: Open Source Solution for Real-Time Peri-Event Time Histogram Based on Open Ephys

**DOI:** 10.3389/fninf.2020.00021

**Published:** 2020-05-20

**Authors:** András Széll, Sergio Martínez-Bellver, Panna Hegedüs, Balázs Hangya

**Affiliations:** ^1^Lendület Laboratory of Systems Neuroscience, Institute of Experimental Medicine, Budapest, Hungary; ^2^Laboratory of Neural Circuitry, Faculty of Medicine and Dentistry, University of Valencia, Valencia, Spain; ^3^János Szentágothai Doctoral School of Neurosciences, Semmelweis University, Budapest, Hungary

**Keywords:** open source, open ephys, peri-event time histogram, optogenetics, behavior, electrophysiology

## Abstract

Single cell electrophysiology remains one of the most widely used approaches of systems neuroscience. Decisions made by the experimenter during electrophysiology recording largely determine recording quality, duration of the project and value of the collected data. Therefore, online feedback aiding these decisions can lower monetary and time investment, and substantially speed up projects as well as allow novel studies otherwise not possible due to prohibitively low throughput. Real-time feedback is especially important in studies that involve optogenetic cell type identification by enabling a systematic search for neurons of interest. However, such tools are scarce and limited to costly commercial systems with high degree of specialization, which hitherto prevented wide-ranging benefits for the community. To address this, we present an open-source tool that enables online feedback during electrophysiology experiments and provides a Python interface for the widely used Open Ephys open source data acquisition system. Specifically, our software allows flexible online visualization of spike alignment to external events, called the online peri-event time histogram (OPETH). These external events, conveyed by digital logic signals, may indicate photostimulation time stamps for *in vivo* optogenetic cell type identification or the times of behaviorally relevant events during *in vivo* behavioral neurophysiology experiments. Therefore, OPETH allows real-time identification of genetically defined neuron types or behaviorally responsive populations. By allowing “hunting” for neurons of interest, OPETH significantly reduces experiment time and thus increases the efficiency of experiments that combine *in vivo* electrophysiology with behavior or optogenetic tagging of neurons.

## Introduction

Neurons are diverse, often referred to as a “zoo.” They are categorized based on their axon, dendrite, soma morphology and connectivity (“m-types”); based on their electrophysiological signatures and activity patterns (“e-types”); and based on their expression of neurotransmitters, neuropeptides, calcium-binding proteins, ion channels and other markers (transcriptomic cell types) (Ascoli et al., 2008; Klausberger and Somogyi, 2008; Sviatkó and Hangya, 2017; Harris et al., 2018; Gouwens et al., 2019). Electrophysiology studies often seek correlations between m-, e-, and genetic types, however, this endeavor is hampered by a paucity of tools that can establish links among these properties, especially during the experiment itself when such tools would be the most useful. Therefore, our overarching goal was to develop a tool that aids real-time identification of cell types in electrophysiology experiments, either defined genetically or by specific response properties.

Optogenetic cell type identification, or tagging, allows identification of genetically defined cell types in extracellular recording by delivering light onto neurons rendered photosensitive by transgenic techniques (Boyden et al., 2005; Lima et al., 2009; Cohen et al., 2012; Kvitsiani et al., 2013; Hangya et al., 2014). However, neurons are often identified during offline analysis, which limits the flexibility and planning of the experiments, resulting in lower number of tagged cells and longer projects. Therefore, our first aim was to provide a tool that allows “hunting” for photosensitive neurons during extracellular recording.

A caveat of optogenetic tagging studies is that light may induce different signals besides spikes, including photoelectric (also called Becquerel) and photovoltaic effects (Kozai and Vazquez, 2015), or exciting too many neuronal elements summing up to population spikes that prevent proper spike sorting. The uneven dispersion of light in brain tissue may lead to artifacts that are hard to remove by offline referencing techniques, as pointed out in previous studies (Cardin et al., 2010; Park et al., 2014; Mikulovic et al., 2016). Most of these potential confounds can be efficiently eliminated by proper control of light intensities delivered into the brain, for which precise online feedback is immensely useful. Therefore, our second goal was to investigate whether a software tool could aid removing light-induced artifacts online to yield a better signal.

Besides genetically defined types, neurons are often characterized by the relation of their firing pattern to external events *in vivo*. For instance, neurons of sensory cortices are categorized by their response to sensory stimuli (Hromádka et al., 2008; Gentet et al., 2012; Hires et al., 2015); conversely, the features of sensory events that activate a given neuron gave rise to the concept of the receptive field (Hubel and Wiesel, 1959; Kilgard and Merzenich, 1998; Ko et al., 2011). Neurons thought to participate in cognitive processing are analyzed with respect to the salience and motivational value of external stimuli (Schultz et al., 1997; Lin and Nicolelis, 2008; Hangya et al., 2015), while neurons on the effector side are correlated with muscle activity and movements (DeLong, 1971). When such representations are sparse, the lack of online identification of the population of interest strongly diminishes experimental throughput. Thus, our third aim was to provide a tool that can visualize alignment of spike firing with external events quasi real-time.

To visualize and quantify the correlation between external events and neural activity, a linear correlation technique called the peri-event or peri-stimulus time histogram is usually applied (Endres et al., 2009; Solari et al., 2018). The PETH is a histogram of relative spike times with respect to the event of interest; thus, it is mathematically equivalent to the cross-correlation of spike and event times. When aiming to study a specific group of neurons, e.g., classically tuned neurons of the primary auditory cortex (Hromádka et al., 2008; Pi et al., 2013) or reward activated neurons of the ventral tegmental area (VTA) (Schultz et al., 1997; Cohen et al., 2012), it is particularly helpful to have a real-time PETH readout during positioning of the recording electrodes.

Therefore, we developed a real-time “online” PETH or OPETH based on the Open Ephys open source data acquisition system (Siegle et al., 2017). It uses the ZMQInterface plugin for distributed messaging and provides a Python interface that receives the data and visualizes peri-event time histograms and evoked waveforms in quasi real-time. We demonstrate that OPETH (i) allows “hunting” for light-sensitive neurons in optogenetic tagging experiments and thus saves significant experimental time; (ii) aids online removal of light-evoked artifacts by adjusting stimulation intensity based on fast feedback, and (iii) increases experimental yield by providing information on neuronal response properties online. In addition, by providing a direct channel of Open Ephys data into Python software, OPETH may serve as a basis for future extensions toward a multipotent Open Ephys – Python interface.

## Methods

In this section we provide a system overview, a description of the Open Ephys ZMQInterface plugin and the signal chain setup and give a detailed presentation of the Python GUI interface for OPETH. Source code is available at https://github.com/open-ephys-plugins/ZMQPlugins and https://github.com/hangyabalazs/opeth.git. Software documentation is available at https://opeth.readthedocs.io.

### System Overview

We provide an overview of data collection and processing to support reproducibility and provide a backbone for more detailed Methods subsections. Animals were implanted with custom-built implants that include Omnetics connectors that can interface with the Intan RHD2000 chip series, compatible with the Open Ephys system (Siegle et al., 2017; Solari et al., 2018). Since Omnetics connectors are the mainstay of neurophysiology implants, our application is not limited by the type of implant and operates with any implementation that interfaces via Omnetics connectors. Data were amplified, digitized at 30 kHz and digitally multiplexed by one or two 32-channel Intan Headstages RHD2132, providing 32- or 64-channel digital recordings. Data were transferred to the Open Ephys (OE) acquisition board by Intan Serial Peripheral Interface (SPI) cables and acquired by the open source, plug-in based Open Ephys Data Acquisition System. This Intan-based OE system is widely used and available, owing to its affordable price and highly flexible applications, capitalizing on a community-based open source strategy.

We chose “zero message queue” (ZMQ or ZeroMQ) for broadcasting data from OE, motivated by its known efficiency and the presence of a partial support within OE. We used a modified ZMQ plug-in^1^ to stream data to external programs and accessed the ZMQ data stream from Python programming language. The OPETH GUI, implemented in Python, visualizes online PETH and evoked waveform plots, providing access to spike discrimination thresholds and other parameters.

The BPod Behavior Control System (Sanworks LLC.) is an open source, microcontroller-based system implementing a finite state machine optimized for low latencies that allow the combination of electrophysiology, optogenetics and animal behavior^2^. We used BPod for real-time behavioral control during animal training. BPod sent TTL pulses at each stimulus onset and reward (water) or punishment (air puff) delivery to synchronize behavioral events with neural recordings.

We used the open source PulsePal stimulator (Sanworks LLC.) to trigger 1 ms square pulses of a blue laser (Sanctity Laser, SSL-473-0100-10TM-D-LED) at 20 Hz with 2 s ON – 3 s OFF duty cycle. The laser light was delivered to the target area by a patch cable (Thorlabs), LC-LC type optical connectors (Thorlabs) and a 50 μm core optical fiber (Laser Components) for optogenetic tagging. TTL pulses were sent both to the blue laser and to Open Ephys to synchronize photostimulation and recording. System components are summarized in Table 1.

**TABLE 1:** List of main components used during the experiments.

**Device**	**Company**
Intan Headstage RHD2132	Intan Technologies
Serial Peripheral Interface cables	Intan Technologies
Open Ephys acquisition board	Open Ephys
Open Ephys Input Output board	Open Ephys
BPod Behavior Control System	Sanwork Inc.
PulsePal	Sanwork Inc.
Blue Laser SSL-473-0100-10TM-D-LED	Sanctity Laser
Custom-built recording microdrive for electrodes and optic fibers	Multiple companies
Silicon probes	Neuronexus

### ZMQInterface Plugin in Open Ephys

Open Ephys is an open source platform for multi-channel electrophysiology experiments (Siegle et al., 2017). The Open Ephys GUI currently works with either of the following hardware: Open Ephys USB acquisition system, Intan RHD2000 Eval Board, Intan RHD Recording Controller, Neuropixels 3a Kintex Acquisition System, Neuropixels 1.0 PXI Acquisition System, White Matter eCube Server, National Instruments USB/PCIe/PXI DAQ boards. This allows Open Ephys to interface with almost any type of commercially available electrodes. In addition, the plugin architecture makes it straightforward to add new data sources. Therefore, Open Ephys can flexibly be implemented for most electrophysiology problems and is widely used in the neuroscience community^3^.

The plugin-based workflow of Open Ephys is designed to facilitate real-time feedback in neuroscience experiments. We used the ZeroMQ interface by Francesco Battaglia (Donders Institute, Radboud University) implemented as a filter plugin to Open Ephys. We updated its API calls to support more recent OE versions, added Windows support and participated in the community effort to make it part of the Open Ephys default distribution. The official ZMQInterface plugin^4^ and its forks were based on Francesco Battaglia’s and our work.

**FIGURE 1:**
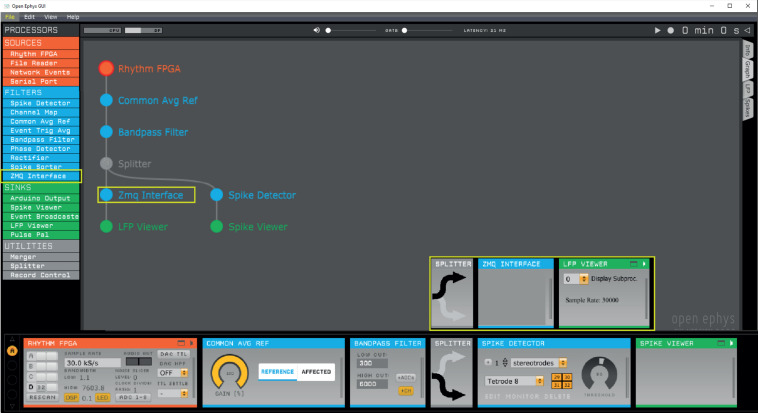
Open Ephys signal chain used to produce the data presented in this study. First, a common average reference filter is applied on the rhythm FPGA data source to remove noise that is common across the electrodes. Next, a band-pass filter rejects low frequency components irrelevant for spike detection. For instance, band-pass filtering between 600 and 6000 Hz enables threshold-based spike detection. Our Python interface implements thresholding itself, therefore, a Spike Detector plug-in should not be included before the ZMQ interface. The ZMQ interface (yellow box), which broadcasts the data to make them available for OPETH, is positioned after the band-pass filter and before the LFP viewer, which visualizes filtered continuous data in the Open Ephys GUI. In a parallel channel, the Open Ephys spike detector filter is applied to allow concurrent visualization of spikes in the Open Ephys GUI.

ZeroMQ is a lightweight network library that simplifies setting up some typical network topologies. The plugin broadcasts recorded data and events that can be subscribed to by external applications through ZeroMQ sockets. The plugin uses a heartbeat mechanism to track which applications are currently connected to the data stream. The data content is dependent on the position of the ZMQ plugin in the signal chain. We use the following signal chain: Rhythm FPGA – Common Average Reference – Bandpass filter – ZMQ Interface – LFP viewer (Figure 1).

The ZMQ Interface plugin opens a ZMQ publisher socket to allow one or more ZMQ clients to subscribe (connect) locally or over the network. Though the system is typically used with a single client connected locally, it is possible to use multiple clients on multiple PCs analyzing the same Open Ephys data source simultaneously with different settings. The ZMQ plugin creates JSON format data packets from the digitized data and event metadata (e.g., timestamp, event channel, number of data channels, and sample count) and sends it over to the client(s). Another socket for event messages and responses is used for heartbeat messages to inform the plugin about the connected clients. The ZMQ plugin is an important component for the OPETH tool, and we made considerable efforts to provide Windows compatibility with recent OE versions.

### Python GUI

Our main goal was to provide online feedback to experimenters on how neural spiking correlates with external events such as photostimulation of neurons during optogenetic experiments or behaviorally relevant events during animal training. Therefore, we developed a graphical user interface in Python based on pyqtgraph^5^ to visualize PETHs aligned to external events during data acquisition in real time. The GUI is compatible with Python 2.7 and Python 3.

At startup, two windows open by default: the main histogram window displaying the online PETH results, and a raw analog data plot for debugging. The “Open new spike win” button in the main window initiates a third view that makes it possible to visually differentiate between spikes and artifacts for a specific channel (see below).

#### Histogram Window

**FIGURE 2:**
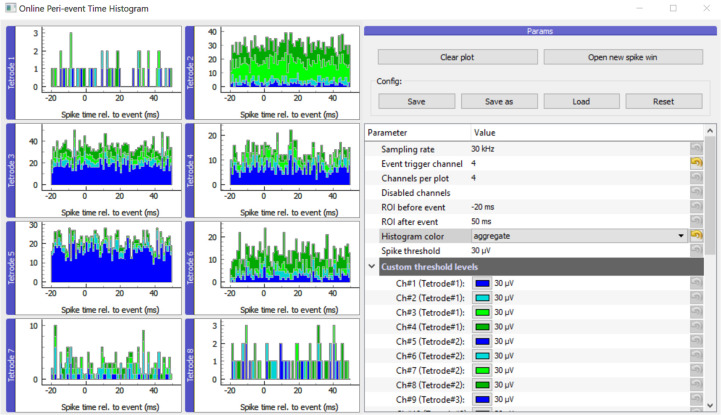
Main GUI of OPETH. The histograms of the different channels or polytrodes are displayed on the left side of the window. The menu on the right side allows changing the parameters and interacting with the GUI. Histogram channels are collected in groups of four by default as for classical tetrode recordings. In order to adapt the plot to other channel configurations, the “Channels per plot” option can be set from 1 to 8 allowing visualization for single electrodes, stereotrodes or silicon probes.

The main GUI window displays histograms, parameters and buttons for handling the configuration and the different plots (Figure 2). Different view modes are available depending on whether the experimenter is interested in events per electrode or single wires/contact sites of multicontact polytrode electrodes (Supplementary Figure S1). To allow flexible use, it is also possible to adjust spike detection threshold levels, sampling rate, event trigger channel and ROI (region of interest: the time window before and after the trigger). As it is a common use case to have multiple experimental projects running in parallel, the parameters can be saved and loaded for each experimental subject in separate ini files. The system remembers the last stored configuration and loads it automatically on startup.

#### Raw Analog Data Window

A real time data viewer window was implemented to display data received directly from Open Ephys, allowing low-level visualization of the output provided by the ZMQ plugin. Since the main purpose of this window is to provide feedback for debugging, channels are auto-scaled and do not provide information on actual voltage levels.

#### Spike Window

**FIGURE 3:**
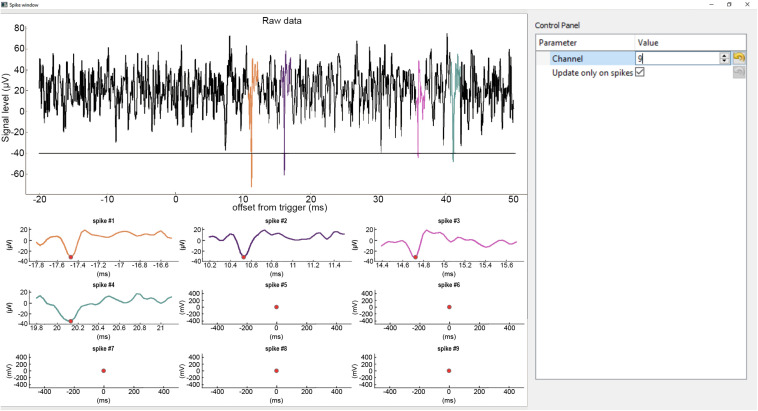
Spike window. Top, continuous raw input data from the selected channel with spikes detected in restricted temporal windows (ROI) aligned to the trigger event (TTLs). The detected spikes are superimposed in color. Horizontal line shows the spike detection threshold. Bottom, zoomed-in windows of the detected spikes. The spike plots display a short segment of data before and after the peak value (red dot) of the spikes (−0.3 ms to +1 ms by default). The same color code is used for the spikes across the plots.

Spike windows can be opened from the main histogram window (Figure 3). Each window displays spikes of a single channel. The purpose of these windows is to show spike waveforms triggered on TTL pulses that can aid the differentiation of light-triggered artifacts from light-evoked neuronal spikes. Multiple Spike windows can be displayed simultaneously, however, this is CPU intensive and opening too many Spike windows will slow down the application.

#### Operation Overview

To provide useful information for developers, we briefly summarize the operation logic of our open source software.

The main window is handled by gui.py, which schedules data reading, spike discrimination, performs histogram calculation and enables the adjustment of parameters. Until the Open Ephys ZMQ plugin connection is established, the GUI displays “Awaiting data.” Once the first chunk of data is received, the exact GUI layout is determined based on the number of channels and the histogram plots are displayed accordingly.

**FIGURE 4:**
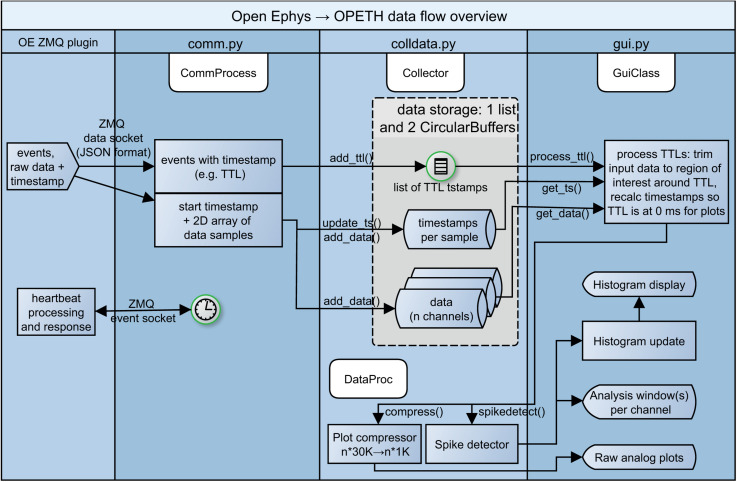
Simplified diagram of data flow. From left to right: heartbeat messages to Open Ephys, events, raw recorded data and heartbeat responses from Open Ephys processed by the communication and data handling layers of OPETH, presented in the GUI.

Input data from OE is received in the form of JSON structures containing the measurement samples and trigger events (handled and parsed by comm.py). Depending on the type of the parsed input data, trigger events and sample data are stored separately (the data flow is managed by the Collector class in colldata.py, see Figure 4). The openephys.py and some of the comm.py interface routines are based on the Python samples created by Francesco Battaglia, while we have developed a circular buffer and data handling methods from scratch as well as the entire visualization UI. The spike detection is performed on the original data as discussed in detail later, whereas the last second of data buffer is downsampled and presented for raw data display.

We note that while the current implementation is single threaded except for ZMQ messaging, it is worth exploring options for multiprocessing in future updates. Figure 4 shows a schematic version of the data flow.

#### Spike Detection

We created a spike discrimination routine to detect extracellular spikes online during recording. This spikedetect() is called whenever a new TTL signal is detected on the currently selected trigger channel. Spike detection is performed for each data channel independently. A simple spike discrimination is performed based on voltage levels within the region of interest (ROI) around the stimulus or timestamped event. Whenever a spike is detected because of exceeding the threshold level, new spikes are not detected until a predefined holdoff time is passed and the voltage level drops below threshold again (known as “censoring”). The implementation works by default with negative threshold levels to allow extracellular spike detection on non-inverted raw voltage data. Please note that overlapping trigger ROIs may result in repeatedly detected spikes in the overlapped region, resulting in a typical “rhythmic” appearance of the plot.

The input data are thresholded by comparing the analog input levels to the threshold parameter. For each spike on the given channel, the starting and final position exceeding the threshold is determined, and the maximum or minimum value of the input data (depending on spike polarity) within this interval gives the spike position (Supplementary Figure S2).

Spike thresholds can be adjusted individually for each channel or for all channels at the same time. It is possible to disable channels or sets of channels from spike detection, e.g., in case of broken channels, listed either comma-separated or with the dash notation (1, 2, 3 or 1–3) in the disabled channels input field. The histograms of the disabled channels are not updated.

### Procedures for *in vivo* Validation Experiments

We performed acute and chronic mouse experiments to test the ability of OPETH to detect neurons of interest, either light-activated in optogenetic tagging experiments or neurons with a specific response to behaviorally salient events. Additionally, we tested whether it is possible to reduce or eliminate light-induced recording artifacts by using OPETH. We also confirmed that OPETH allows significant savings in invested experimental time. We provide procedural details of these experiments for reproducibility purposes below. The experimental results are detailed thereafter in the Results section.

#### Animals

Electrophysiological, optogenetic and behavioral data showed in this study were obtained from 13 adult mice (3 BAC-Vglut2-IRES-Cre C57Bl/6J and 3 PV-IRES-Cre FVB/AntFx males, 3 vGAT-IRES-Cre Bl6Fx and 4 ChAT-IRES-Cre B6129F1 females). All experiments were approved by the Committee for Scientific Ethics of Animal Research of the National Food Chain Safety Office and were performed according to the guidelines of the institutional ethical code and the Hungarian Act of Animal Care and Experimentation (1998; XXVIII, section 243/1998, renewed in 40/2013) in accordance with the European Directive 86/609/CEE and modified according to the Directives 2010/63/EU.

#### Surgery and Virus Injection

Mice were anesthetized with an intraperitoneal injection of ketamine-xylazine (0.166 and 0.006 mg/kg, respectively). The scalp was shaved and disinfected (Betadine) and local anesthetics was applied subcutaneously (Lidocaine). The mouse was positioned in the stereotaxic frame and the eyes were protected with eye ointment (Laboratories Thea). The skin was removed above the calvaria and the skull was cleaned; the head of the animal was leveled using Bregma and Lambda (Konsman, 2003) and lateral points equidistant from the sagittal suture.

In 12 of the Cre animals, a cranial window was opened in order to access the medial septum (MS) with a 10° lateral angle (MS 10°, antero-posterior +0.90 mm, lateral, 0.90 mm). An adeno-associated virus vector allowing Cre-dependent expression of channelrhodopsin2 [AAV 2/5. EF1a.Dio.hChR2(H134R)-eYFP.WPRE.hGH] was injected into the MS at 3.95, 4.45, and 5.25 mm depth from brain surface (200 nl at each depth). The skin was sutured; local antibiotics (Neomycin) and a subcutaneous injection of analgesic (Buprenorphine 0.1 mg/kg) were applied.

In a ChAT-Cre animal, a craniotomy was performed above the horizontal nucleus of the diagonal band of Broca of the basal forebrain (HDB, antero-posterior 0.75 mm, lateral 0.60 mm) and the same virus was injected into the HDB at 5.00 and 4.70 mm depth from brain surface (300 nl at each depth). Additional holes were drilled above the parietal cortex for ground and reference. The surface of the skull was covered with a thin layer of Super-Bond C&B (Sun Medical) and a custom-built microdrive (Kvitsiani et al., 2013; Hangya et al., 2015) with 8 tetrodes was implanted in the targeted area. The microdrive-skull junction was protected with Kwik–Cast sealant (World Precision Instruments). The microdrive was secured to the skull with dental acrylic resin (Lang Dental). A titanium headbar was also attached to the skull to allow headfixation. Analgesic and antibiotics were applied as above. Mice were allowed to recover for 10 days, receiving subcutaneous injections of analgesic (Buprenorphine 0.1 mg/kg) and local application of antibiotics (Neomycin) as necessary.

#### Anesthetized Recordings

Two weeks after the virus injection the Vglut2-Cre animal was anesthetized with an i.p. injection of 20% urethane (Sigma-Aldritch, 0.007 ml/g body weight). The depth of anesthesia was evaluated by pinching the paw or ear of the animal. When there were no reflexes elicited by the pinching, the throat was shaved and topical lidocaine was applied. A tracheotomy was performed in order to sustain a constant airflow (Moldestad et al., 2009). The animal was placed in a stereotaxic frame and, after opening the skin and leveling the skull, trephine holes were made above the MS (silicon probe MS 10°, antero-posterior, +0.90 mm, lateral, 0.90 mm; optic fiber MS 5° contralateral, antero-posterior, +0.90 mm, lateral, −0.50 mm), the hippocampus (silicon probe HPC, antero-posterior, −2.20 mm, lateral, 1.50 mm) and two above the cerebellum for reference electrodes. A Neuronexus A1 × 32 6 mm-50-177-CM32 silicon probe was placed in the hippocampus at 2.20 mm depth from brain surface, and a Neuronexus Buzsaki32-H32_21 mm probe was lowered to the dorsal boundary of the MS at a 10° lateral angle (3.95 mm from brain surface). Reference electrodes for both probes were placed in the cerebellum and ground electrode was placed in the spinotrapezius muscle. A 200 μm core optic fiber was lowered 500 μm above the shanks of the MS probe. The MS probe and the optic fiber were lowered in 100 μm steps for recording, spanning the entire depth of the MS. Extracellular data were collected by the Open Ephys data acquisition system, digitized at 30 kS/s. Each recording session consisted of an optical tagging period of 2 min, followed by a baseline period of 5 min. Three consecutive repetitions of 1 min tail pinch-induced theta activity followed by 1 min control recording were applied, finishing the recording session with another 2 min long optical tagging period. After each recording session, the MS probe and optic fiber were lowered 100 μm.

#### Head-Fixed Recordings and Behavioral Procedures

Once fully recovered, the ChAT-Cre animal implanted with a microdrive was trained on a head-fixed auditory cued outcome task implemented in a go/no-go paradigm. Briefly, the animal was water restricted for 3 days. On the fourth day, the animal was head-fixed in the behavioral environment (Solari et al., 2018), where after a few free water delivery trials, a go tone (10 kHz, 50 dB, 1 s) was presented. Licking during the tone resulted in the release of a 3 μl water droplet as reward. Once the animal was familiarized with this paradigm, a second tone (4 kHz, 50 dB, 1 s) was introduced, predicting the delivery of an air puff (duration, 200 ms). In the final task, a balanced mixture of the two tones were randomly interleaved in which the 10 kHz tone predicted expected reward in 80% of trials, unexpected punishment in 10% of the trials and omission in the remaining 10% of trials; the 4 kHz tone predicted expected punishment 65% of trials, unexpected reward in 25% of the trials and omissions in the remaining 10%. Extracellular data were collected during task performance by the Open Ephys data acquisition system, digitized at 30 kS/s.

#### Data Analysis

Offline data analysis was performed using built-in and custom-built Matlab (Mathworks) scripts. Spikes were detected by a threshold crossing algorithm similar to online spike sorting. After detecting the spikes on all channels belonging to the same tetrode, spike times were merged, sorted, and a censoring period was applied to prevent repeated detection of the same spike. When censoring, the algorithm kept the largest spike in the censoring window. Spikes were manually sorted into putative neuronal clusters based on amplitude (peak-to-valley), waveform energy and first principal component features using the MClust 3.5 software (A. D. Redish). L-ratio (<0.05) and isolation distance (>20) were taken as cluster quality measures (Schmitzer-Torbert et al., 2005).

Neurons expressing ChR2 were identified by optogenetic tagging (Lima et al., 2009; Kvitsiani et al., 2013; Pi et al., 2013). Significant light activation was assessed by the Stimulus-Associated spike Latency Test (SALT)^6^ (Kvitsiani et al., 2013).

Neural data used for generating the figures and data analysis code are available at https://gin.g-node.org/hangyabalazs/OPETH_validation_data and https://github.com/hangyabalazs/CellBase.

The OPETH software has been assigned the RRID of SCR_018022.

## Results

OPETH was designed with the purpose of providing real-time feedback during electrophysiology experiments. To this end, it visualizes histograms and raw spike waveforms aligned to external events communicated by digital logic signals, as explained in section Methods. We hypothesized that such a tool would allow online detection of neurons of interest: either photostimulated cells in optogenetic tagging experiments or neurons with desired response patterns.

To test this, we performed two experiments and compared online detection by OPETH with *post hoc*, offline detection carried out after concluding the experiments. Offline analysis allowed more precise detection due to less constraints on filter design for spike detection, optimized detection based on combined information from all contact sites/wires within an electrode, spike sorting and statistical testing of significant spiking responses. In the first experiment, we performed silicon probe recordings and optogenetic tagging from the medial septum (MS) area of the basal forebrain of anesthetized mice to gauge the value of OPETH for optogenetic cell type identification. In the second experiment, we trained awake behaving mice on an associative learning task. We performed chronic recordings from the horizontal nucleus of the diagonal band of Broca (HDB) and tested whether OPETH is capable of detecting reinforcement-responsive neurons. We demonstrated the presence of punishment-activated HDB neurons during the recording online with OPETH, and later confirmed this result by offline analyses.

### Real-Time Optogenetic Tagging

**FIGURE 5:**
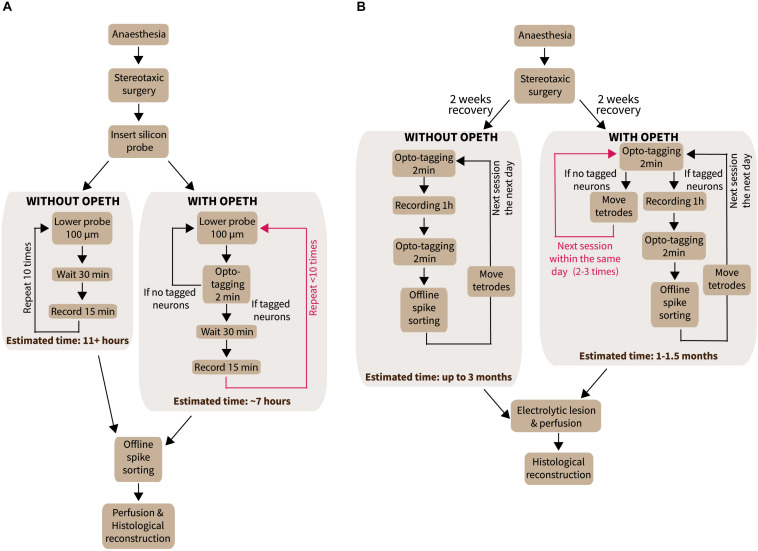
Experiments performed with OPETH. **(A)** Workflow of acute anesthetized experiments with or without OPETH. OPETH saved more than 4 h per experiment on average. **(B)** Workflow of chronic recording experiments in awake behaving mice with or without OPETH. Using OPETH in chronic tetrode recording experiments, we were able to make several tagging sessions within the same day, without having to record and process all data, thus allowing us to take faster decisions and save up to 2 months of training and recording.

Optogenetic tagging allows the identification of neuron types in extracellular recordings performed in transgenic animals. For instance, we use optogenetic tagging in acute anesthetized experiments to investigate the role of different genetically defined types of MS neurons in the genesis of neural oscillations and network synchrony (Wang, 2002; Hangya et al., 2009; Buzsáki and Moser, 2013; Figure 5A). We hypothesized that the yield of such experiments could greatly be increased if the presence of optogenetically tagged neurons would be established online during the recording.

**FIGURE 6:**
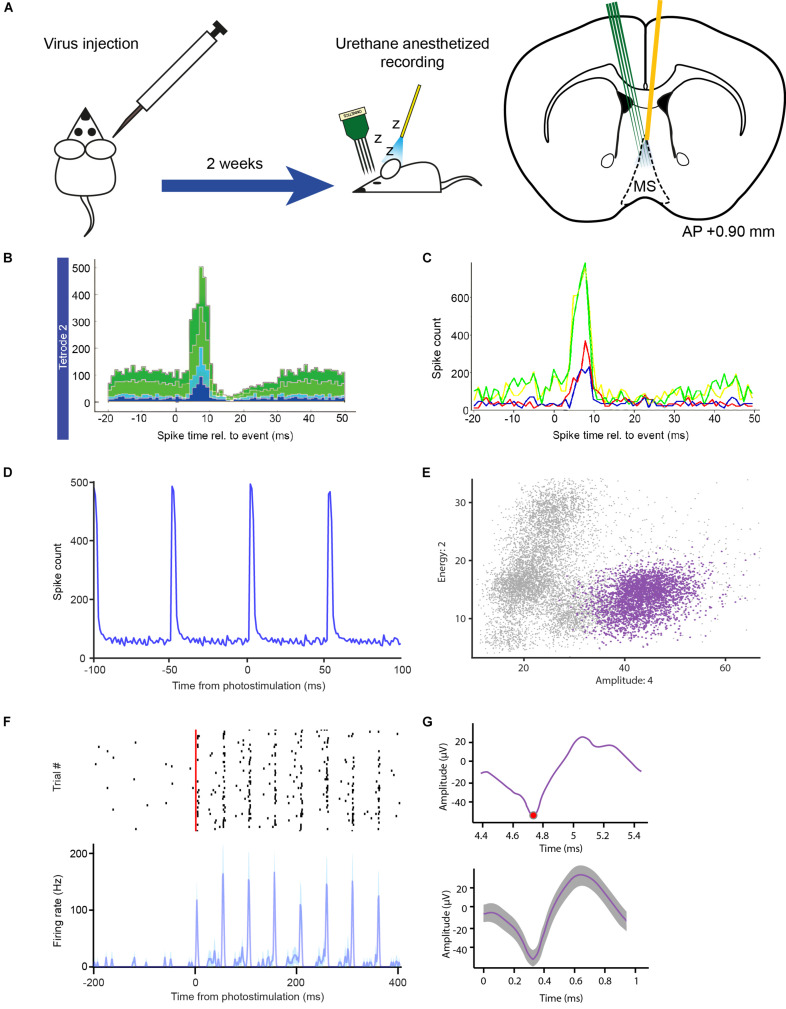
*In vivo* optogenetic tagging experiment for testing OPETH. **(A)** Transgenic animals were virus injected and, after a 2 weeks recovery period, acute recordings were performed under urethane anesthesia, in which a 32-channels silicon probe and an optic fiber were inserted in the MS in order to record light-activated units. **(B)** OPETH *Histogram Window (“aggregate view”)* shows neural responses to laser pulses in a Vglut2-Cre mouse expressing channelrhodopsin2 (1 ms bin size). **(C)** OPETH “*Channel view”* of the same recording was used to determine the most responsive channel of the tetrode of interest. In this view, histograms are calculated separately for single channels (arbitrary color code). **(D)** The offline peri-event time histogram calculated from all spikes of the same tetrode (unsorted data of all neurons) confirmed the presence of strong light-evoked activity. **(E)** Spikes from the same tetrode were sorted into putative single neurons using MClust 3.5. The plot shows an optotagged neuron in feature space (purple). Note the relatively small deviations around the cluster centroid, characteristic to real spikes in contrast with light-evoked artifacts. **(F)** Spike raster (black, spike times) and peri-event time histogram of the sorted MS single neuron example aligned to the onset of the laser pulse train (red), from the channel selected via OPETH (resolution, 1 ms). **(G)** Top, raw light-evoked spike in the OPETH *Spike window*. Bottom, average spike shape of the same neuron analyzed offline.

Therefore, in our first experiment, we used OPETH for online optogenetic tagging of medial septal glutamatergic neurons in an *in vivo* acute anesthetized experiment. A BAC-Vglut2-IRES-Cre mouse was injected with a viral construct allowing Cre-dependent expression of the light sensitive channelrhodopsin2 protein in glutamatergic MS neurons (Figure 6A). After a recovery period of 2 weeks that also allowed sufficient virally driven channelrhodopsin2 expression, the mouse was anesthetized with urethane and placed in a stereotax. A 32-channel linear silicon probe was placed in the hippocampus for local field potential recordings and a 32-channel four-shank silicon probe was lowered into the MS for extracellular recording of MS units. In addition, an optic fiber was placed in the MS above the recording probe to deliver laser light for photostimulation.

Once the probe was lowered to the desired position, a quick tagging session was performed in order to assess the presence of putative light activated cells. Laser-triggered responses of MS neurons were monitored by the OPETH *Histogram Window* throughout the experiment (Figures 6B,C). If the presence of light-triggered spikes was confirmed (see also the “*Eliminating photostimulation artifacts with OPETH*” section), the tissue was left to stabilize for 30 min and then the recording session was started. If no photoactivation was observed during the tagging session, the probe was lowered again and a new tagging session was performed. The process was repeated throughout the dorso-ventral extent of the MS. This protocol allowed us to “hunt” for optogenetically identified glutamatergic MS neurons and remove the potential confounds arising from photostimulation-related electrical artifacts, thus increasing the efficiency of the experiment (Figure 5A).

After concluding the experiment, we performed offline peri-event time histogram analysis (Figure 6D). Similar to the online detection procedure, spikes were detected from each recording channel. However, offline analysis allowed the application of a non-causal Butterworth filter, while online filtering typically involves causal finite impulse response filters. Additionally, censoring (the process of eliminating multiple detections of the same spike) was optimized across tetrode channels by keeping the largest spikes within the censoring windows, which process typically yielded 10–20% more detected spikes. Binary time series were generated from the spiking and photostimulation event point processes at 1 ms resolution and Matlab’s built-in xcorr.m function was used to derive the PETH aligned to the onset of the photostimulation pulses. This confirmed the presence of light responses on the same tetrodes as shown by OPETH (Figure 6D).

Next, we performed spike sorting using MClust 3.5 software (A.D. Redish; Schmitzer-Torbert et al., 2005; Hangya et al., 2015). Putative single neurons that exhibited significant light responses tested by the Stimulation-Associated spike Latency Test (SALT, *p* < 0.001; Kvitsiani et al., 2013) were identified on the same tetrode that showed the presence of light-evoked spikes in OPETH (Figures 6E,F). Moreover, neurons that showed optogenetic response online and offline could be matched based on spike shape features (Figure 6G). Finally, spike sorting of three recording sessions revealed significantly light-activated neurons recorded by the same tetrodes as indicated during the online assessment by OPETH (*n* = 10, *p* < 0.001).

### Eliminating Photostimulation Artifacts With OPETH

Light can induce changes of electric potential in the material of metal electrodes through photoelectric mechanisms (Kozai and Vazquez, 2015; Mikulovic et al., 2016). Additionally, dense expression of light-sensitive actuators combined with high light intensity may result in simultaneous activation of multiple neurons and potentially different neuronal elements including both fibers and somata, leading to a population spike (Cardin et al., 2010; Cohen et al., 2012; Kvitsiani et al., 2013; Mikulovic et al., 2016). Since such population spikes prevent proper spike sorting and thus optogenetic identification of light-activated neurons, they can be considered as “biological artifacts.” Common to these undesired signals is that they are sensitive to reducing stimulation light intensity. However, light intensities below a threshold will fail to activate neurons, leading to false negatives. We expected that online feedback helps finding the optimal stimulation level that still activates individual neurons close to the recording electrodes but avoids evoking artifacts. We note that Open Ephys provides a Spike View that displays waveforms triggered by threshold crossings. Nevertheless, this view shows all spikes and not only light-evoked ones, which are usually the minority; therefore, it has limited use in providing the necessary feedback for stimulation adjustments.

**FIGURE 7:**
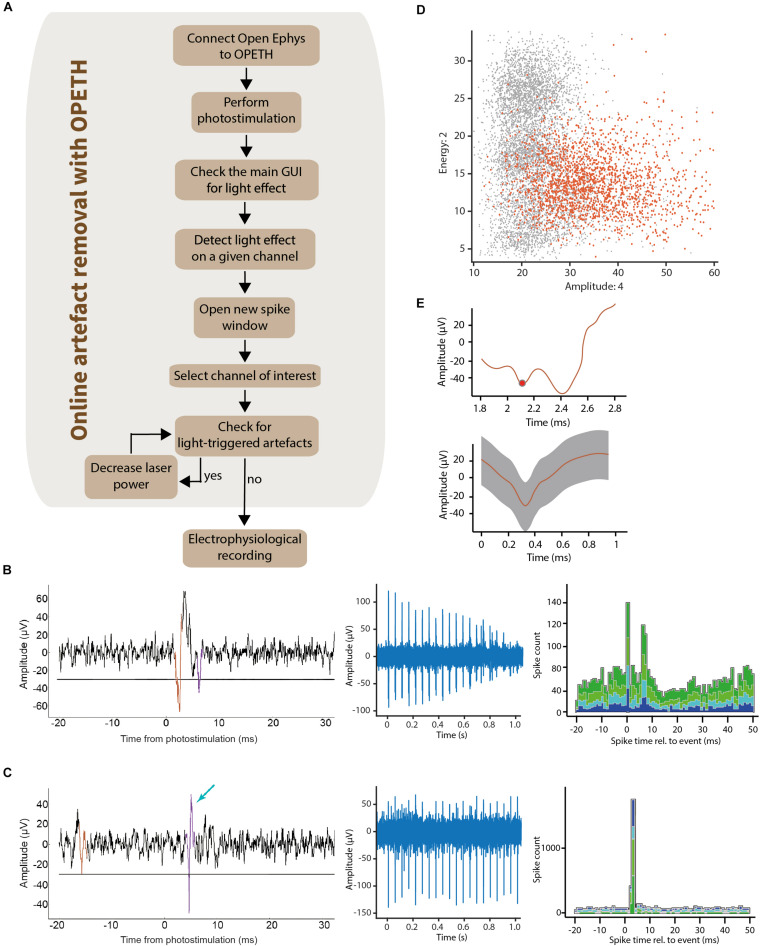
Removing light-triggered artifacts from electrophysiological recordings. **(A)** It was possible to determine the channels affected by photostimulation by checking the main GUI during optogenetic tagging sessions. The raw signal was visualized using the “Open new spike window” button of the GUI. This allowed the experimenter to control laser intensity until light-triggered artifacts disappeared from the plots. **(B)** Left, raw data of the light-evoked artifact aligned to photostimulation (waveform superimposed in color). Middle, decreasing the laser intensity lead to decreasing amplitude of the artifacts. Right, the corresponding OPETH histogram showed a bimodal PETH caused by the “W shape” of the light-evoked artifacts. **(C)** Left, raw data of a light-evoked spike (waveform superimposed in color, indicated by blue arrow). Middle, the all-or-none spikes did not show amplitude changes upon decreasing stimulation intensities. Right, corresponding online histogram in the main OPETH window. **(D)** Waveforms plot in feature space showed a high dispersion of the light-evoked artifacts (orange). **(E)** Top, raw signal of the light-evoked artifact in the OPETH *Spike window*. Bottom, average artifact waveform in the offline analysis.

Therefore, we designed a simple protocol that allows artifact elimination by using OPETH (Figure 7A). Once putative light-evoked spikes were detected in the main GUI on one of the electrodes, the *Channel view* was used, which allowed us to determine the precise recording channel that registered short-latency responses to light activation. Detecting the “responsive electrode” first and then zooming in on the “responsive channel” was more effective than viewing and evaluating every single channel separately. A *Spike Window* for the affected channel was then opened and monitored while adjusting light intensity levels to avoid artifacts. The waveform of light-induced artifacts was clearly different from spikes to trained experimenters: artifacts were broader, more variable (especially on the later descending phase) and lacked the characteristic shape of extracellularly recorded action potentials (Figures 7B–E). Unlike “all-or-none” spikes (Figure 7C), undesired artifacts typically scaled with light intensity (Figure 7B). Therefore, if artifacts were detected, then the stimulation intensity was reduced. This procedure was repeated until artifacts were eliminated.

### Estimation of Experimental Time Saved by OPETH During Anesthetized MS Recordings

Time optimization is a key feature in the design and development of experiments. In the previous sections, we have demonstrated the capabilities of OPETH for online assessment of the effects of photostimulation, allowing active “hunting” for responsive neurons while avoiding recording artifacts. Every time optogenetic effects were not detected online, we lowered the electrode by 100 μm and repeated the online detection (Figure 5A). Once OPETH signaled the presence of light-activated units, we waited 30 min for the brain tissue around the electrodes to settle (typical in these types of experiments) and then performed 15 min long recording sessions. We noticed that this procedure saved significant amounts of experimental time.

In the following, we provide an estimate of the time saved by OPETH during our acute anesthetized recordings. In our original experimental design, we recorded ten times from the MS, lowering the silicon probe 100 μm between each recording, which allowed us to map the entire dorso-ventral aspect of the MS. The surgery preceding the recordings, including a delicate procedure of stereotaxic implantation of fragile silicon probes, could take up to 3 h from the onset of urethane anesthesia. Each MS recording consisted of 30 min waiting time to allow the brain tissue to stabilize after moving the probe, followed by 15 min of recording, which sums up to a total of 7.5 h of recording including the waiting times. With post-recording surgical procedures, the experimental time could exceed 11 h in a single experimental session.

We analyzed the time saved by OPETH in 12 anesthetized MS experiments using different genetic mouse models. Excluding the pre-recording surgery, the waiting and recording times of 12 animals was estimated around a total of 90 working hours (12 × 7.5 h). By using OPETH to only record MS positions that showed online light response, the cumulative waiting plus recording time was 58.5 h (Table 2), saving a total 31.5 h of work. We estimated that each recording required around 30 min of the experimenter’s time to be fully analyzed. Therefore, OPETH was able to save an additional 21 h by avoiding the analysis of 42 sessions of the theoretically possible 120 recordings that would have been collected without using OPETH. Altogether, OPETH thus saved a total of 52.5 h of working time.

**TABLE 2:** Duration of the anaesthetized MS surgeries assisted by OPETH.

**Animal ID**	**Experiment time (omitting surgery) (h)**
PV-Cre 1	7.5 h
PV-Cre 2	5.25 h
PV-Cre 3	5.25 h
VGat-Cre 1	6 h
VGat-Cre 2	4.5 h
VGat-Cre 3	3.75 h
VGlut-Cre 1	5.25 h
VGlut-Cre 2	5.25 h
VGlut-Cre 3	3.75 h
ChAT-Cre 1	4.5 h
ChAT-Cre 2	3.75 h
ChAT-Cre 3	3.75 h
Total time	58.5 h

### Real-Time Peri-Event Time Histogram in Behaving Mice

In addition to optogenetic tagging, OPETH enables online tracking of neural responses to behaviorally relevant external events such as cue stimuli and reinforcement. To demonstrate this, we next tested OPETH’s ability to detect neuronal activity changes during a head-fixed go/no-go task in an awake behaving mouse (Figure 5B).

**FIGURE 8:**
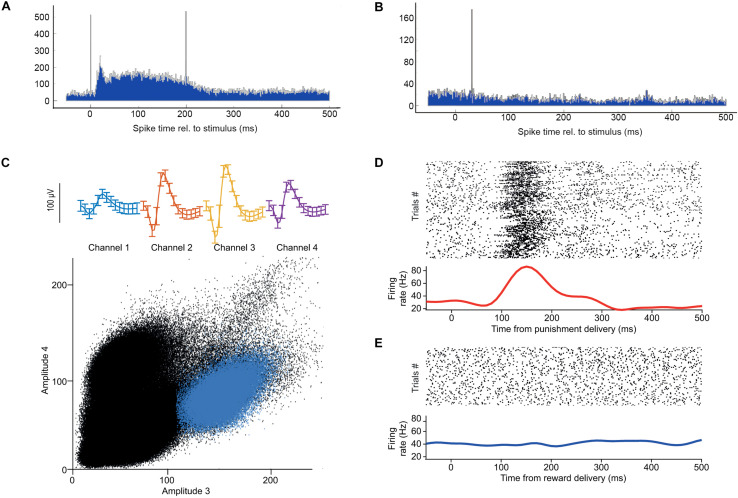
Neuronal responses to behaviorally relevant events detected real-time by OPETH. **(A)** OPETH online histogram aligned to delivery times of negative behavioral feedback (punishment). OPETH indicated an increase in neuronal activity in response to punishment delivery (vertical lines indicate artifacts due to valve opening and closing). **(B)** OPETH online histogram aligned to delivery times of positive behavioral feedback (reward). No neuronal response was detected after reward delivery. **(C)** After offline analysis, punishment-responsive neurons were detected on the tetrodes indicated online by OPETH. The plot shows spike shape (top) and cluster projection (bottom) of an example punishment-activated reward-unresponsive neuron after offline spike sorting. **(D)** Spike raster and PETH aligned to the onset of punishment delivery for the same neuron. **(E)** Spike raster and PETH aligned to the onset of reward delivery for the same neuron.

A mouse was fully trained on a head-fixed auditory cued outcome task, in which two pure tones of different pitch signaled different outcome probabilities, predicting either likely reward (water) vs. surprising punishment (a puff of air) or vice versa. Two different TTL pulses were sent to the Open Ephys I/O board every time reward or punishment was delivered, allowing OPETH to visualize the neuronal response to each of the behavioral outcomes. The mouse performed a total of 254 trials in a single recording session. Throughout this session, the *Histogram Window* of OPETH clearly showed a neuronal response to punishment on most tetrodes (Figure 8A), while no response to reward delivery could be detected (Figure 8B). We noted that this punishment response was already detectable after the first few punished trials, showing the sensitivity of detection by OPETH.

After the recording session, we performed offline spike detection and spike sorting as described in the “Real-time optogenetic tagging” section (Figure 8C). We visualized the activity of each neuron by aligning the spike times to reward and punishment time stamps in raster plots and peri-event time histograms. This offline analysis confirmed the presence of neurons that responded selectively to punishment, as expected based on the online feedback by OPETH (Figures 8D,E). Figures 8C–E shows a well-isolated neuron that responded with an increase of firing after air puff punishment, but not after water reward.

### Matching Between Online and Offline Light-Evoked Activity

Performing multiple chronic recordings in awake behaving mice allowed us to quantify the correspondence between online assessment of light-effects and offline detection of photosensitive neurons.

We analyzed 18 recording sessions of a ChAT-Cre mouse implanted with 8 moveable tetrodes (a total of 144 tetrode recordings; the four channels of the tetrodes were not compared individually). A tetrode was considered to show light effects online if short-latency light-evoked spikes were present on any of the four tetrode wires after artifact elimination, judged by the experimenter using OPETH *Histogram Window* and *Spike Window* during the experiment. After the experiments, offline detection was performed in Matlab using a superior Butterworth-filter typically applied for offline spike detection. The censoring algorithm was optimized using information from all four tetrode wires, as described above. Offline peri-event time histograms were calculated based on all spikes from the entire recording, resulting in increased statistical power compared to online analysis. Therefore, offline analysis theoretically allowed higher precision due to slightly more sophisticated, albeit CPU-intensive algorithms, and the benefit of using the entire recording. This could result in light-responses detected offline but not online (insufficient statistical power for online detection), which constituted the “false negatives” of OPETH. At the same time, we hypothesized that insufficient artifact elimination with OPETH might result in online but not offline detection, which would lead to “false positive” OPETH detections.

To test these, we assessed the degree of overlap between online detection by OPETH and offline analysis. During the sessions, OPETH detected light responses in 46/144 tetrode recordings. Offline analysis showed that 37/46 of these detections were true positives, while 9/46 detections were false positives. OPETH did not detect light responses in 98/144 recordings. Of these, 96/98 were true negatives, while 2 were false negatives assessed by offline analysis (Table 3). Overall, OPETH showed a statistical sensitivity, or true positive rate of 94.87% and a specificity, or true negative rate of 91.43%.

**TABLE 3:** Matching between online and offline detection of light-evoked activity.

**Session**	**OPETH light-activated tetrodes**	**Offline light-activated tetrodes**	**True positives**	**False positives**	**False negatives**	**True negatives**
1	3	2	2	1	0	5
2	3	3	3	0	0	5
3	4	2	2	2	0	4
4	8	7	7	1	0	0
5	0	1	0	0	1	7
6	2	2	2	0	0	6
7	1	1	1	0	0	7
8	3	2	2	1	0	5
9	2	2	2	0	0	6
10	2	2	2	0	0	6
11	4	4	4	0	0	4
12	5	4	4	1	0	3
13	2	2	2	0	0	6
14	1	2	1	0	1	6
15	1	0	0	1	0	7
16	1	1	1	0	0	7
17	2	0	0	2	0	6
18	2	2	2	0	0	6
Total	46	39	37	9	2	96

### Latency Measurements of the ZMQInterface

OPETH may also provide a steppingstone for future Python-based real-time applications, including closed loop experiments using triggers based on neuronal activity. To facilitate this, we performed latency measurements of the Open Ephys-based system. We used the ZMQInterface plugin and only certain parts of OPETH (“stub”), because the current application structure would not support quick responses and short latencies.

**FIGURE 9:**
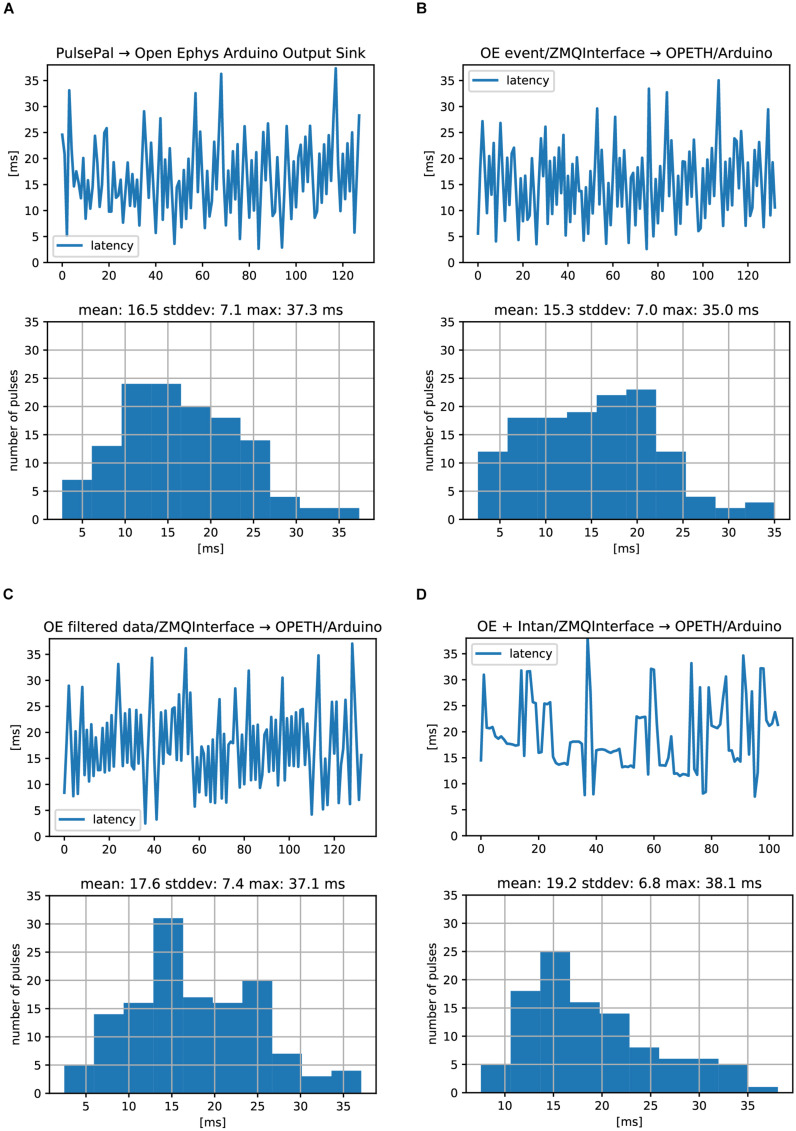
Open Ephys analog and digital signal chain latencies. Oscilloscope delays were measures between an input TTL pulse and the generated response TTL. **(A)** Serially performed latency measurements (top) and histogram of latency values (bottom) corresponding to the setup in Supplementary Figure S3A. **(B)** Same measurements for the setup in Supplementary Figures S3B. **(C)** Same measurements for the setup in Supplementary Figures S3C. **(D)** Same measurements for the setup in Supplementary Figure S3D. No differences between digital **(A,B)** and analog **(C)** signal processing were found. ZMQInterface **(B–D)** did not introduce long delays.

In the first setup, a straightforward TTL loopback test was performed, where an Open Ephys Arduino Output sink was directly triggered by the digital input of the Open Ephys acquisition board. This resulted in 16.5 ± 7.1 ms (mean ± SD; note that values may be affected by time domain quantization) delay compared to the input signal (Supplementary Figure S3A and Figure 9A).

The second setup also used the digital input to the Open Ephys acquisition board, but we replaced the Arduino Sink with the ZMQInterface plugin, which broadcasted the events to an OPETH stub (Supplementary Figure S3B). This stub generated output pulses by USB-UART byte writes to trigger the Arduino Uno’s output TTL pulse, using a custom Arduino firmware. First, we found that PyZMQ protocol latency was 1.2 ± 0.15 ms (mean ± SD), measured by handshake messages between a ZMQ Request/Response pair. A test for the heartbeat message between the OPETH stub and OE ZMQInterface plugin resulted in slightly longer delays of 1.4 ± 0.2 ms, with rare (<1%) outliers in the range of 4–12 ms. In conclusion, one-way ZMQ messaging overhead for locally transmitted short packets was typically on the order of 1 ms, but OE introduced relatively rare, an order of magnitude longer outliers. Second, Arduino Uno USB-UART delays were measured similarly to ZMQ heartbeat messages. We chose the highest available baud rate of 2 MBaud/s and tested both directions for getting a response to the “heartbeat” character. PC to Arduino and back took 3 ± 0.4 ms (mean ± SD), while starting from Arduino side took 2.3 ± 1.2 ms, measured at 4 μs resolution. We expected that replacing the Arduino Sink of the first setup with the ZMQInterface and OPETH stub triggering an Arduino TTL via USB-UART will add some extra latency, but its timing result of 15.3 ± 7.0 ms was well within error margins (and in the particular case even faster; Figure 9B).

As a digital input trigger could have considerably less latency than analog buffered data packets that need to be filtered before being sent to the consumer, we tested the analog input of the Open Ephys board and extended the OPETH stub with data thresholding in the third setup (Supplementary Figure S3C). In this mode, the ZMQInterface plugin sent data arrays instead of trigger events. We found a mean latency of 17.6 ms, which was only 2 ms longer than the previous setup (Figure 9C). Repeating the measurement after including Common Average Reference and Bandpass filters in OE did not alter the mean delay.

The last setup emulated our test bench used in normal experiments. The input TTL signal was connected to a Universal Signal Mouse and the attenuated pulses were recorded by an Intan headstage (32 channels) connected directly to the OE acquisition board, omitting the OE I/O board from the circuit (Supplementary Figure S3D). Analog latency measurements showed 19.2 ± 6.8 ms average delays (Figure 9D).

We would like to note that we did not test the impact of data rate and used Windows 10 OS without adjusting process priorities. Experiments with more strict latency requirements would necessitate the use of specific external hardware and/or suitable real-time operation systems. Also note that most delay measurements showed some periodicity, suggesting that measurements were not fully independent. This likely does not affect the maximum latencies but closed-loop experiments that demand high temporal precision may require more precise delay assessments. However, a comprehensive Open Ephys closed-loop benchmark was beyond the scope of this study.

## Discussion

Real-time analysis while performing electrophysiology recordings is important to guide decisions during the experiment. Here we described OPETH, an open source online tool for visualizing peri-event time histograms. We demonstrated that it is useful when conducting optogenetic tagging or behavioral experiments combined with single cell or multiunit recording and showed that it is possible to achieve significant time savings by using OPETH. OPETH is based on Open Ephys, an open source data acquisition system including software (Siegle et al., 2017). It is implemented in Python, which, by providing an intuitive and multi-purpose development environment, gained widespread popularity in neuroinformatics. Thus, OPETH may provide a potential seed for an Open Ephys to Python interface that could greatly reduce the threshold for developing Open Ephys plugins by obviating the need of C++ coding.

### Open Source

There is an increasing number of open source tools in neuroscience, which is also paralleled by an increased awareness of the open source movement in general (Gleeson et al., 2017). An important example is Open Ephys, enabled by the development of Intan chips that allowed an affordable upscaling of electrophysiology experiments. Combined with open source tools for behavior control (Sanders and Kepecs, 2012), stimulation (Sanders and Kepecs, 2014) and full behavioral environments (Erlich et al., 2011; Devarakonda et al., 2016; Solari et al., 2018), this array of recent affordable, modular, flexible and easy to scale tools has changed the way electrophysiology experiments are performed. We provide OPETH as a new member of this family that parallels the richness of features of commercial solutions (e.g., Neuralynx Histogram Display^7^), at the same time available to the entire neuroscience community.

Some tools targeted similar objectives as OPETH and the ZMQInterface plugin. The PSTH^8^ plugin of Open Ephys was introduced in 2014/2015 but it was soon discontinued. It had a limited user interface compared to OPETH and was more integrated into OE. Recently, a MatlabInterface^9^ (formerly MatlabEngine) plugin was introduced, bearing a resemblance to the ZMQInterface plugin. It may be more recommended for Matlab integration, as we experienced hard to resolve hangups in Matlab using the ZMQInterface. A third option for extending OE functionality into high-level programming languages is the PythonPlugin^10^. This solution integrates well with Open Ephys and it has features ZMQInterface and OPETH do not support, but development iterations are less flexible and need somewhat deeper knowledge (e.g., it is not possible to modify code without interrupting a recording session in Open Ephys, and Cython is required for PythonPlugin development).

### Application: Real-Time *in vivo* Cell Type Identification

In neuroscience, many of the key insights were gained by recording the electrical activity of neurons (Sviatkó and Hangya, 2017). An instructive example was the mapping of basal ganglia neurons while monkeys were engaged in a variety of behavioral tasks (DeLong, 1971; DeLong et al., 1984). DeLong and colleagues performed basic linear convolution-based data analysis in the form of raster plots and peri-event time histograms, which still remains the mainstay of systems neuroscience. Eventually, these results lead to the Deep Brain Stimulation surgeries during which stimulating electrodes are lowered to the subthalamic nucleus of the basal ganglia in Parkinson’s patients, largely alleviating their otherwise often crippling motor impairments. However, the lack of proper tools to identify the great diversity of anatomically, histochemically and hodologically defined cell types of the basal ganglia *in vivo* stalled further progress (Sviatkó and Hangya, 2017).

This was first overcome by glass pipettes that allowed filling of the recorded cells by applying current pulses, called juxtacellular recording (Pinault, 1996). Then, the recent advent of imaging and optogenetic techniques (Ghosh et al., 2011; Park et al., 2015; Shin et al., 2017) opened the way to high-throughput cell type identification in awake, behaving rodents (Al-Hasani et al., 2015; Miller et al., 2019; Wang et al., 2019). This necessitates the development of new software tools aligned to this task, enabling significant increases of experimentation efficiency. OPETH provides a way of online tracking cellular responses to light flashes, in order to optogenetically identify those neurons that respond with short latency. This allows determining whether the target area has been reached, and good quality recordings of identified units can be performed. Therefore, by enabling “hunting” for neurons of interest, this tool can efficiently increase the yield of optogenetic tagging experiments (Figure 5).

### Application: Online Tracking of Response Properties to Behaviorally Relevant Events

Peri-event time histograms usually represent the first-pass analysis of neuronal activity of behaving animals (Endres et al., 2009; Shimazaki and Shinomoto, 2010). We have demonstrated here that this first-level analysis can be performed online, providing immediate feedback on the responsiveness of the recorded population. This may be especially useful when looking for neurons with a particular response profile, or cell types that can be identified by features of their responses. Since areas may differ significantly in the proportion of neurons responding to different sensory cues, OPETH may also allow the rough identification of target areas. Other applications include online receptive field mapping (Froemke et al., 2007) or precise localization along the frequency axis of auditory cortical tonotopy maps (Hromádka et al., 2008).

## Conclusion and Future Directions

By providing online access to event-aligned linear data statistics, OPETH also opens the door to more advanced online analysis. For instance, dopaminergic neurons in VTA may be identified by principal component analysis of their PETH aligned to reward and reward-predicting cues, as demonstrated by Cohen et al. (2012) and later applied by other labs (Takahashi et al., 2016). Therefore, adding this analysis to OPETH may allow online identification of dopaminergic cells without performing optogenetics. Other examples include online analysis of delay activity in working memory tasks or correlating neuronal firing with reward expectations or prediction errors.

The modified ZMQInterface plugin enables having an extended framework implemented in Python in the future, allowing direct implementation of Python-based data analysis tools that include spike sorting (Pachitariu et al., 2016), raster plot and waveform analysis, filtering and analysis of brain oscillations (Oliphant, 2007; Garcia and Fourcaud-Trocmé, 2009; Muller et al., 2015). PETH calculations can be integrated in many analytical workflows, allowing the optimization of online data analysis. Moreover, a cornucopia of mathematical algorithms commonly used in neuroscience, including convolution, Fourier-transformation, wavelet transform and various statistical approaches are available through NumPy, PyWavelet and other distributions. Therefore, a viable Open Ephys – Python interface will make development of new online analysis more accessible to all neuroscientists.

ZMQInterface and OPETH also allow future implementation of closed-loop protocols. Closed-loop approaches are gaining momentum as part of experimental procedures (El Hady, 2016) as well as in clinical applications (Ghasemi et al., 2018). Closed-loop neuronal recording in behavioral tasks has been used for assessing the role of the mouse primary visual cortex during navigation (Saleem et al., 2013), enhancing spatial navigation skills of mice by optical manipulation of the hippocampal theta oscillation cycles (Siegle and Wilson, 2014), determining the causal involvement of sharp wave ripple events in learning (Rangel Guerrero et al., 2018) and to control Drosophila feeding behavior (Moreira et al., 2019). OPETH can be used as a programmable open-source tool for closed-loop paradigms based on the detected neuronal activity, allowing high-precision automatic control of the desired output.

## Data Availability Statement

All datasets generated for this study are available at https://gin.g-node.org/hangyabalazs/OPETH_validation_data.

## Ethics Statement

The animal study was reviewed and approved by the Committee for Scientific Ethics of Animal Research of the National Food Chain Safety Office.

## Author Contributions

BH conceived the project. AS developed the OPETH software. SM-B and PH performed the validation experiments and data analysis. SM-B, AS, and PH prepared the figures. BH, AS, and SM-B wrote the manuscript with inputs from PH.

## Conflict of Interest

The authors declare that the research was conducted in the absence of any commercial or financial relationships that could be construed as a potential conflict of interest.

## Footnotes

1 https://github.com/NeuroNetMem/ZMQInterface

2 https://sanworks.io/shop/products.php?productFamily=bpod

3 https://open-ephys.org

4 https://github.com/open-ephys-plugins/ZMQPlugins, ZMQInterface folder

5 www.pyqtgraph.org

6 http://kepecslab.cshl.edu/salt.m

7 https://neuralynx.com/software/category/sw-acquisition-control

8 https://open-ephys.atlassian.net/wiki/spaces/OEW/pages/23265293/PSTH

9 https://github.com/medengineer/MatlabInterface

10 https://github.com/NeuroNetMem/PythonPlugin

## Supplementary Material

FIGURE S1 Different visualization modes in the main GUI window. The plotting mode can be controlled separately in each histogram window by setting “Histogram color.” **(A)** “Flat” view. Events from all single wires/contact sites of the multiwire/multicontact electrode are summed and displayed as a single histogram. **(B)** “Aggregate” view. Events are still summed within an electrode, but single wires/contact sites are overlaid on the histogram in different colors. **(C)** “Channels” view. Histograms are calculated for individual wires/contact sites within multiwire multicontact electrodes and plotted as separate, colored lines. If single channels are to be compared, it is recommended to use the “channels” histogram view.

FIGURE S2 Schematics of spike detection. Key variables for spike detection based on threshold crossings are indicated, including spike position (peak) and censoring period (also called “dead time”). Note that sample count does not correspond to default 30 kS/s.

FIGURE S3 Measurement setups for latency quantifications. The measurement setup consisted of a PulsePal (Sanworks) generating 5V TTL pulses (1 ms on/500 ms period time). We connected the PulsePal output channel #1 to channel #1 of an Open Ephys IO board, which was connected to an Open Ephys acquisition board. The OE board was managed from a Windows 10 PC, which generated response TTLs for the closed-loop protocols using an Arduino Uno. The delay between the input and output pulses were measured on a PicoScope 3205D, its GUI running on the same PC. The scope measurements were performed creating an A+B Math channel and utilizing the DeepMeasure feature of Picoscope. **(A)** The digital trigger generated a pulse via an Arduino Output sink and an Arduino Uno board. For this, the StandardFirmata firmware was loaded to the Arduino and TTL pulses were generated on pin #13. **(B)** The ZMQInterface broadcasted the trigger and OPETH stub generated pulse via an Arduino. **(C)** Filtered analog data was broadcasted to the OPETH stub for threshold detection. **(D)** The IO board was replaced by a Universal Signal Mouse (Neuralynx) and an Intan Headstage for analog measurements. This test was performed on a different computer than the previous measurements.
