# Supplementary figures and images for: OPETH: Open Source Solution for Real-Time Peri-Event Time Histogram Based on Open Ephys
Source: Front Neuroinform. 2020 May 20;14:21. doi: 10.3389/fninf.2020.00021 (PMC7251067; doi:10.3389/fninf.2020.00021)

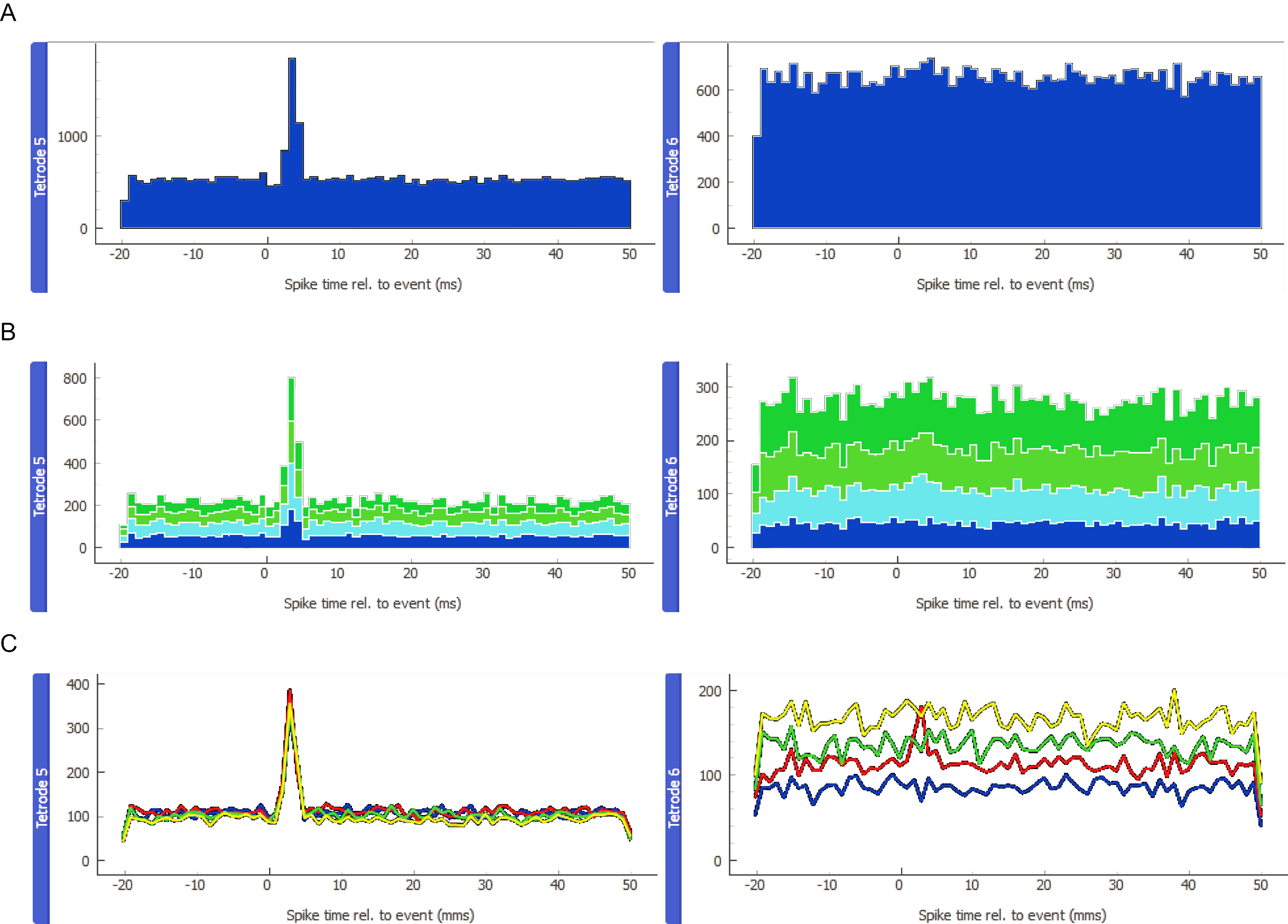

Supplement: FIGURE S1 — Different visualization modes in the main GUI window. The plotting mode can be controlled separately in each histogram window by setting “Histogram color.” (A) “Flat” view. Events from all single wires/contact sites of the multiwire/multicontact electrode are summed and displayed as a single histogram. (B) “Aggregate” view. Events are still summed within an electrode, but single wires/contact sites are overlaid on the histogram in different colors. (C) “Channels” view. Histograms are calculated for individual wires/contact sites within multiwire multicontact electrodes and plotted as separate, colored lines. If single channels are to be compared, it is recommended to use the “channels” histogram view. [file Image_1.JPEG]

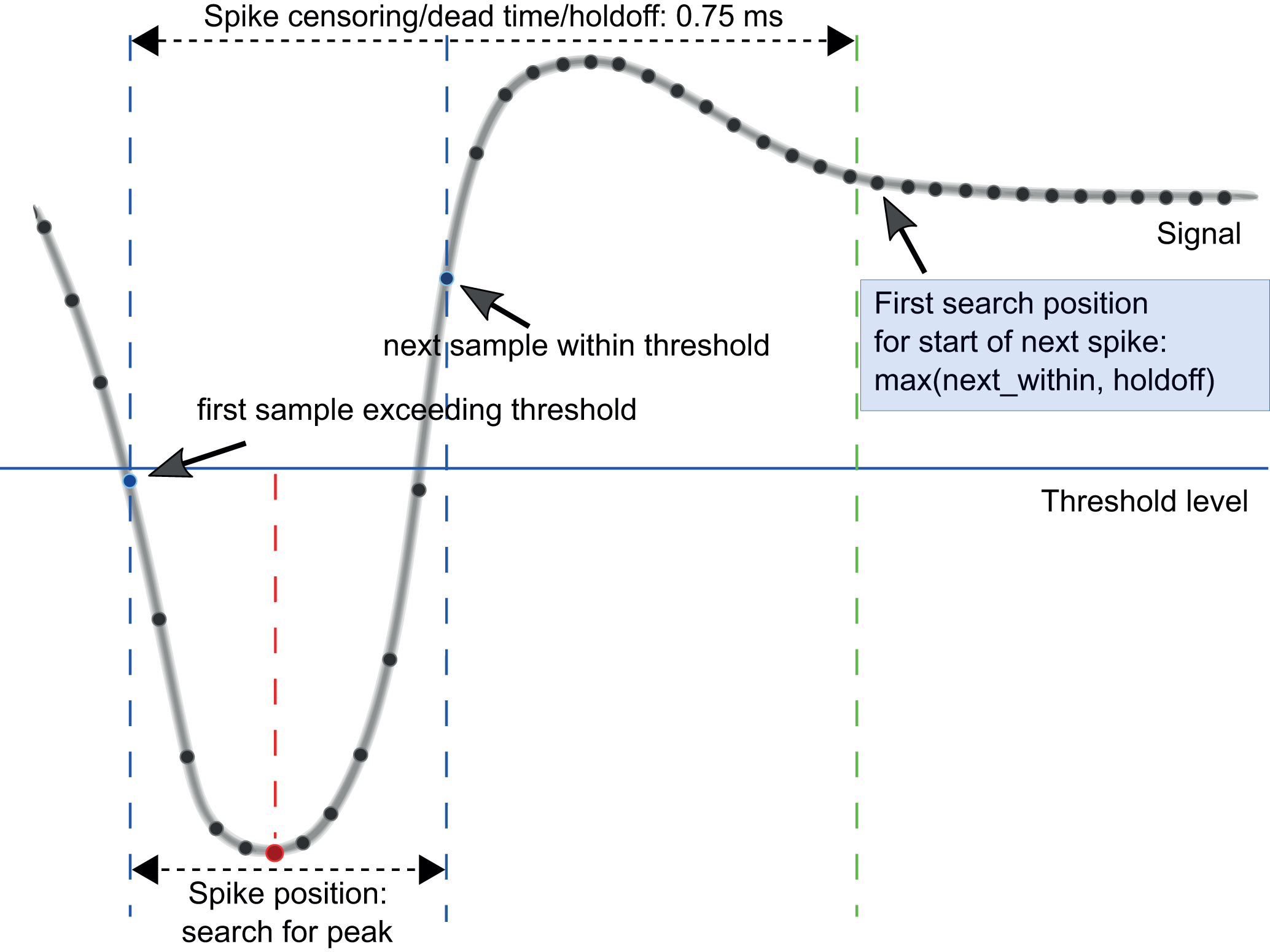

Supplement: FIGURE S2 — Schematics of spike detection. Key variables for spike detection based on threshold crossings are indicated, including spike position (peak) and censoring period (also called “dead time”). Note that sample count does not correspond to default 30 kS/s. [file Image_2.JPEG]

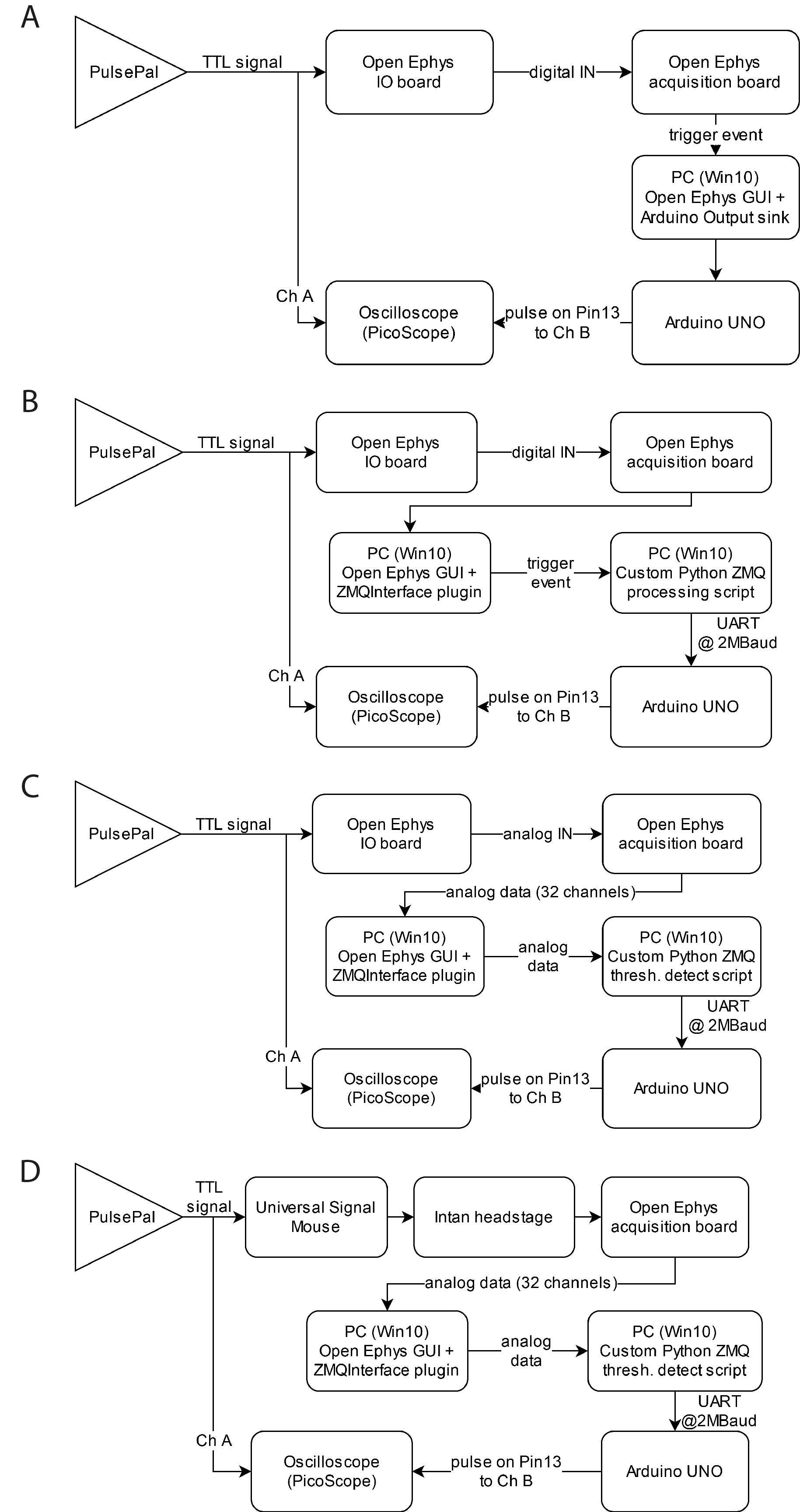

Supplement: FIGURE S3 — Measurement setups for latency quantifications. The measurement setup consisted of a PulsePal (Sanworks) generating 5V TTL pulses (1 ms on/500 ms period time). We connected the PulsePal output channel #1 to channel #1 of an Open Ephys IO board, which was connected to an Open Ephys acquisition board. The OE board was managed from a Windows 10 PC, which generated response TTLs for the closed-loop protocols using an Arduino Uno. The delay between the input and output pulses were measured on a PicoScope 3205D, its GUI running on the same PC. The scope measurements were performed creating an A+B Math channel and utilizing the DeepMeasure feature of Picoscope. (A) The digital trigger generated a pulse via an Arduino Output sink and an Arduino Uno board. For this, the StandardFirmata firmware was loaded to the Arduino and TTL pulses were generated on pin #13. (B) The ZMQInterface broadcasted the trigger and OPETH stub generated pulse via an Arduino. (C) Filtered analog data was broadcasted to the OPETH stub for threshold detection. (D) The IO board was replaced by a Universal Signal Mouse (Neuralynx) and an Intan Headstage for analog measurements. This test was performed on a different computer than the previous measurements. [file Image_3.JPEG]
